# A New Risk Scheme to Predict Ischemic Stroke and Other Thromboembolism in Atrial Fibrillation: The ATRIA Study Stroke Risk Score

**DOI:** 10.1161/JAHA.113.000250

**Published:** 2013-06-21

**Authors:** Daniel E. Singer, Yuchiao Chang, Leila H. Borowsky, Margaret C. Fang, Niela K. Pomernacki, Natalia Udaltsova, Kristi Reynolds, Alan S. Go

**Affiliations:** 1 Clinical Epidemiology Unit, Massachusetts General Hospital, Boston, MA (D.E.S., Y.C., L.H.B.); 2 Department of Medicine, University of California San Francisco, San Francisco, CA (M.C.F., A.S.G.); 3 Division of Research, Kaiser Permanente of Northern California, Oakland, CA (N.K.P., N.U., A.S.G.); 4 Department of Research and Evaluation, Kaiser Permanente of Southern California, Pasadena, CA (K.R.); 5 Department of Epidemiology, University of California San Francisco, San Francisco, CA (A.S.G.); 6 Department of Biostatistics, University of California San Francisco, San Francisco, CA (A.S.G.); 7 Department of Health Research and Policy, Stanford University School of Medicine, Palo Alto, CA (A.S.G.)

**Keywords:** anticoagulants, atrial fibrillation, risk score, stroke

## Abstract

**Background:**

More accurate and reliable stroke risk prediction tools are needed to optimize anticoagulation decision making in patients with atrial fibrillation (AF). We developed a new AF stroke prediction model using the original Anticoagulation and Risk Factors in Atrial Fibrillation (ATRIA) AF cohort and externally validated the score in a separate, contemporary, community‐based inception AF cohort, ATRIA–Cardiovascular Research Network (CVRN) cohort.

**Methods and Results:**

The derivation ATRIA cohort consisted of 10 927 patients with nonvalvular AF contributing 32 609 person‐years off warfarin and 685 thromboembolic events (TEs). The external validation ATRIA‐CVRN cohort included 25 306 AF patients contributing 26 263 person‐years off warfarin and 496 TEs. Cox models identified 8 variables, age, prior stroke, female sex, diabetes mellitus, heart failure, hypertension, proteinuria, and eGFR<45 mL/min per 1.73 m^2^ or end‐stage renal disease, plus an age×prior stroke interaction term for the final model. Point scores were assigned proportional to model coefficients. The c‐index in the ATRIA cohort was 0.73 (95% CI, 0.71 to 0.75), increasing to 0.76 (95% CI, 0.74 to 0.79) when only severe events were considered. In the ATRIA‐CVRN, c‐indexes were 0.70 (95% CI, 0.67 to 0.72) and 0.75 (95% CI, 0.72 to 0.78) for all events and severe events, respectively. The C‐index was greater and net reclassification improvement positive comparing the ATRIA score with the CHADS_2_ or CHA_2_DS_2_‐VASc scores.

**Conclusions:**

The ATRIA stroke risk score performed better than existing risk scores, was validated successfully, and showed improvement in predicting severe events, which is of greatest concern. The ATRIA score should improve the antithrombotic decision for patients with AF and should provide a secure foundation for the addition of biomarkers in future prognostic models.

## Introduction

Atrial fibrillation (AF) is a common arrhythmia that increases the risk for ischemic stroke by 4‐ to 5‐fold.^1^ Oral anticoagulant therapy is highly effective at reducing stroke risk in patients with AF but raises the risk of major bleeding.^2–3^ Leading clinical practice guidelines^4–7^ recommend a risk‐based approach to the anticoagulation decision in AF, usually based on the CHADS_2_^8^ or CHA_2_DS_2_‐VASc^9^ stroke risk scores. However, these risk scores have only moderate ability to predict which patients will have a stroke.^10–11^ To improve estimation of stroke risk, we developed and internally validated a new prediction model using the distinctively large experience of the Anticoagulation and Risk Factors in Atrial Fibrillation (ATRIA) AF cohort. We then externally validated the resulting new risk score in the recently assembled contemporary inception AF cohort, the ATRIA‐Cardiovascular Research Network (ATRIA‐CVRN).

## Methods

### Cohort Assembly

Assembly and validation of the ATRIA nonvalvular AF cohort have been described in detail previously.^12^ In brief, we used health plan databases to identify adult members of Kaiser Permanente Northern California who had an outpatient AF diagnosis between July 1, 1996, and December 31, 1997. We included all patients ≥18 years old with either 2 or more outpatient AF diagnoses (ICD‐9 code 427.31) or 1 outpatient AF diagnosis with ECG validation. The date of the first AF diagnosis was considered the patient's index date. Warfarin exposure was based on a validated algorithm using information from pharmacy and laboratory databases.^13^ Of the 13 559 patients in the ATRIA cohort, 9217 took warfarin at some point during follow‐up, contributing 33 497 person‐years of observation, and 10 927 contributed some time off warfarin during follow‐up. We used only person‐time off warfarin to develop our stroke risk model. In the Results section we analyze all person‐time off warfarin. The results comparing risk scores were very similar when we restricted the analysis to the 4342 patients who did not take warfarin at any point during follow‐up (data not shown).

### Patient Baseline Characteristics

Clinical characteristics for patients in the ATRIA cohort were ascertained by searching inpatient, outpatient, laboratory, and pharmacy databases for the relevant ICD‐9 codes, medications, or lab values within the 5 years prior to the patient's index date (specific codes available by request) as well as administrative databases.^12–13^ The Kaiser Permanente longitudinal diabetes registry was also used to identify patients with diabetes mellitus. These approaches for identifying comorbid conditions from electronic databases have been previously validated against a review of samples of patient medical records. Crude agreement ranged between 78% and 96% for individual risk factors.^12^ Methods for ascertaining proteinuria and estimated glomerular filtration rate (eGFR) are provided in a prior publication.^14^

### Outcome Event Identification

ATRIA cohort members were followed from their index date through September 2003. Ascertainment of outcome events has been described previously.^13^ Follow‐up was censored at the date of the outcome event, death (ascertained via hospital databases, health plan reporting, Social Security Administration vital status files, and the California state death registry), or health plan disenrollment. Medical records of potential outcome events were reviewed by 2 physician reviewers.^13^ Ischemic stroke was defined as sudden onset of a neurologic deficit lasting >24 hours and not attributable to other identifiable causes.^13^ Other thromboembolic events were considered valid if they met the following criterion: sudden occlusion of an artery to a visceral organ or extremity documented by imaging, surgery, or pathology and not attributable to concomitant atherosclerosis or other etiology. Events first occurring in‐hospital or resulting from periprocedural complications were excluded. Each event was assigned a modified Rankin score indicating the level of disability at the time of hospital discharge.^15^

### ATRIA‐CVRN Cohort

We externally validated the ATRIA score in the separate ATRIA‐CVRN AF cohort. The ATRIA‐CVRN study cohort is made up of 33 247 patients from Kaiser Permanente Northern California and also Kaiser Permanente Southern California aged 21 or older with incident atrial fibrillation (AF) or atrial flutter first diagnosed between January 2006 and June 2009 with confirmation by ECG or physician diagnosis in the electronic medical record. Validating diagnoses of AF included ≥1 inpatient diagnosis or ≥2 outpatient diagnoses. Unlike the ATRIA cohort, the ATRIA‐CVRN cohort did not exclude patients with mitral stenosis or a history of a valve replacement in the mitral or aortic positions; such patients account for 1.5% of the ATRIA‐CVRN cohort.

Determination of baseline features, warfarin use, and outcome event occurrence and adjudication for the ATRIA‐CVRN cohort followed the same approach as for the ATRIA cohort except that for the ATRIA‐CVRN cohort emergency department visits that did not result in hospital admission could still count as valid outcome events. Patients were followed from their index date through June 2009. Follow‐up was censored at the date of the outcome event, death, or health plan disenrollment.

### Statistical Analyses

#### Model derivation and internal validation in the original ATRIA cohort

We used a split‐sample approach to develop and internally validate the new stroke risk score. Patients with any periods off warfarin were randomly divided into a derivation cohort that accounted for approximately two thirds of person‐years contributed and a validation cohort that had the remaining one third of person‐years. We preselected 10 candidate predictor variables previously reported as stroke risk factors in atrial fibrillation: older age, female gender, prior ischemic stroke, diabetes, heart failure, hypertension, coronary artery disease (CAD), peripheral arterial disease (PAD), urine dipstick proteinuria, and low eGFR or end‐stage renal disease (ESRD) requiring dialysis.^8–9,14,16–17^ In addition, we considered total white blood cell count as an inflammatory marker and an episode of herpes zoster.^17–18^ Age was categorized as <65, 65 to 74, 75 to 84, or ≥85 years old, and total white blood cell count was categorized as <8000, 8000 to 9999, or ≥10 000 per microliter. eGFR was dichotomized at ≥45 versus <45 mL/min per 1.73 m^2^ or ESRD. On the basis of univariate analysis results in the derivation cohort, we also tested an additional interaction term of age by prior stroke. All risk factor values were updated over the follow‐up period using the last value carried forward method. The follow‐up periods that did not have a preceding laboratory measurement going back as far as 5 years were considered normal (ie, WBC<8000/μL, eGFR ≥60 mL/min per 1.73 m^2^, and no proteinuria). These imputed normal values accounted for 3.5% of the person‐years for WBC, 2.8% of the person‐years for eGFR, and 22.2% of the person‐years for proteinuria.

To minimize false‐positive variable selection,^19^ we constructed 1000 bootstrap samples based on the derivation two‐thirds cohort. For each sample, we used a time‐updated Cox proportional hazards model^20^ with the backward elimination method to determine predictors significant at the 0.05 level after variable selection. Variables consistently chosen in >60% of bootstrap samples were included in the final model. Traditional measures of model fit and their confidence intervals were calculated for both the derivation cohort and the validation cohort, including the c‐index^21^ for discrimination and the goodness‐of‐fit statistic for calibration.^22^ To generate a risk score, we assigned points to each variable proportional to its regression coefficients rounded to the nearest integer.

#### Comparison of ATRIA score with CHADS_2_ and CHA_2_DS_2_‐VASc scores

We compared the discrimination capacity of the full‐range ATRIA stroke risk score with the full‐range CHADS_2_ and CHA_2_DS_2_‐VASc scores in the entire ATRIA off‐warfarin cohort using the c‐index.^21^ In addition, we collapsed the ATRIA stroke risk score into 3 categories on the basis of observed annual thromboembolic (TE) rates: low (TE rate <1.0%), moderate (TE rate 1.0 to <2.0%), and high (TE rate ≥2.0%). These rate cut points were based on a formal decision analysis.^23^ We compared the performance of the ATRIA 3‐category scheme to the CHADS_2_ and CHA_2_DS_2_‐VASc published 3‐category schemes both in terms of the c‐index and net reclassification improvement.^8–9,24^

#### Score performance predicting severe events and in primary prevention

We assessed the performance of our model in predicting severe TE events defined as those with a Rankin score ≥3 at hospital discharge (representing moderate to severe disability) or death within 30 days following the event. In addition, we tested the risk score in the primary prevention subset (ie, those individuals with no history of prior stroke).

#### Validation of ATRIA risk score performance in an external cohort

All model performance assessments were replicated in the separate ATRIA‐CVRN cohort. All analyses were conducted using SAS software, version 9.3 (SAS Institute, Inc, Cary, NC). The study was approved by the institutional review boards of the collaborating institutions. Waiver of informed consent was obtained because of the nature of the study.

## Results

### Construction of the ATRIA Risk Score

From the original ATRIA cohort of 13 559 AF patients, we accumulated 32 609 person‐years off warfarin contributed by 10 927 patients during the follow‐up period. The median time off warfarin was 2.4 years (range, <1 month to 7.2 years). We validated 685 TE events within this cohort (643 ischemic strokes and 42 other TE events) for an annualized rate of 2.1%. In the two‐thirds derivation cohort, there were 7284 patients with a total of 21 739 person‐years of follow‐up and 456 TE events. In the one‐third validation cohort, there were 3643 patients, 10 870 person‐years of follow‐up, and 229 TE events.

**Figure 1.**
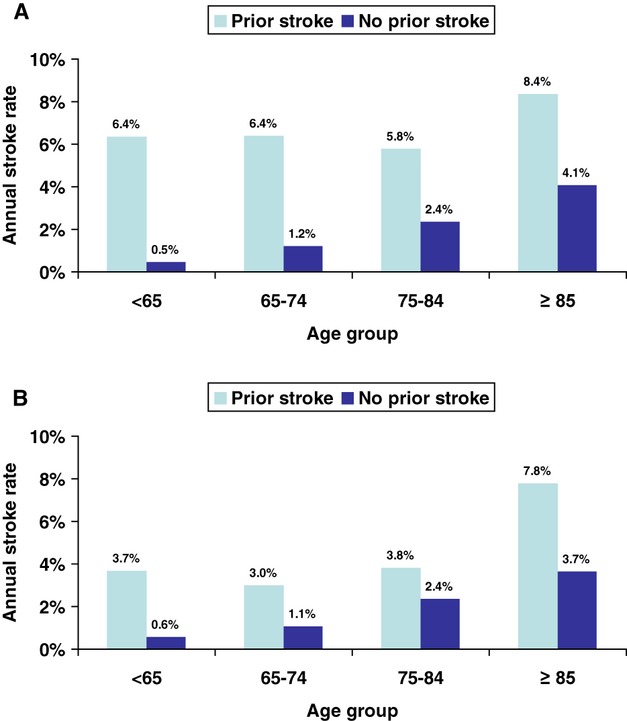
A, Annual stroke rates by age category in the Anticoagulation and Risk Factors in Atrial Fibrillation (ATRIA) off‐warfarin cohort using the following cutoffs for age groups: <65, 65 to 74, 75 to 84, and ≥85 years. B, Annual stroke rates by age category in the ATRIA‐CVRN off‐warfarin cohort using the following cutoffs for age groups: <65, 65 to 74, 75 to 84, and ≥85 years.

In univariate analysis, all the selected predictor variables were significantly related to stroke risk except for a history of herpes zoster (Table 1). Thromboembolism risk increased monotonically across all 4 age categories, including a near‐doubling of risk for those ≥85 years compared with those 75 to 84 years old. The effect of age was particularly strong among the large group of patients without a prior stroke. However, the effect of older age was muted in patients with a history of prior stroke. Patients with a prior stroke in the 3 younger age categories all had high rates of subsequent stroke, about 6% per year. Patients ≥85 years with a history of stroke had the highest rate of stroke, >8% per year. This interaction of the effects of age and prior stroke was statistically significant (*P*<0.0001; Figure 1A).

**Table 1.** Univariate Analysis of Potential Clinical Predictors of Thromboembolism for the Entire ATRIA Study Cohort

Variable	Level	Person‐Years	% Person‐Years	Rate per 100 Person‐Years	Hazard Ratio (95% CI)
Age category	<65	7752	23.8%	0.57	Ref
	65 to 74	8421	25.8%	1.41	2.38 (1.69 to 3.37)
	75 to 84	11 115	34.1%	2.58	4.46 (3.24 to 6.12)
	≥85	5322	16.3%	4.42	8.14 (5.91 to 11.2)
Prior ischemic stroke	N	30 992	95.0%	1.87	Ref
	Y	1618	5.0%	6.61	3.28 (2.66, 4.04)
Age×prior ischemic stroke	<65, stroke	126	0.4%	6.35	12.1 (5.42 to 26.8)
	65 to 74, stroke	314	1.0%	6.38	11.7 (6.75 to 20.1)
	75 to 84, stroke	760	2.3%	5.79	11.1 (7.14 to 17.2)
	≥85, stroke	418	1.3%	8.37	17.2 (10.74 to 27.7)
	<65, no stroke	7626	23.4%	0.47	Ref
	65 to 74, no stroke	8107	24.9%	1.22	2.49 (1.70 to 3.64)
	75 to 84, no stroke	10 355	31.8%	2.35	4.90 (3.45 to 6.95)
	≥85, no stroke	4903	15.0%	4.08	9.06 (6.36 to 12.9)
Sex	M	18 648	57.2%	1.54	Ref
	F	13 961	42.8	2.85	1.86 (1.60 to 2.17)
Heart failure	N	24 089	73.9%	1.67	Ref
	Y	8520	26.1%	3.32	1.91 (1.64 to 2.23)
Hypertension	N	14 306	43.9%	1.57	Ref
	Y	18 303	56.1%	2.52	1.67 (1.42 to 1.96)
Coronary artery disease	N	23 279	71.4%	1.84	Ref
	Y	9331	28.6%	2.74	1.47 (1.26 to 1.72)
Peripheral arterial disease	N	31 656	97.1%	2.07	Ref
	Y	953	2.9%	3.04	1.47 (1.01 to 2.13)
Herpes zoster	N	32 123	98.5%	2.10	Ref
	Y	486	1.5%	2.47	1.34 (0.75 to 2.40)
Diabetes mellitus	N	27 066	83.0%	1.92	Ref
	Y	5543	17.0%	2.99	1.57 (1.31 to 1.86)
White blood cell count, per μL	<8000	21 818	66.9%	1.85	Ref
	8000 to <10 000	6657	20.4%	2.36	1.26 (1.05 to 1.52)
	≥10 000	4134	12.7%	3.00	1.58 (1.29 to 1.93)
Estimated glomerular filtration rate, mL/min per 1.73 m^2^	≥60	20 920	64.2%	1.44	Ref
	45 to 59	6888	21.1%	2.61	1.79 (1.49 to 2.16)
	30 to 44	3525	10.8%	4.37	3.00 (2.47 to 3.64)
	15 to 29	968	3.0%	3.82	2.61 (1.85 to 3.68)
	<15 or ESRD	308	0.9%	3.90	2.58 (1.45 to 4.57)
Urine dipstick proteinuria	None or trace	27 660	84.8%	1.83	Ref
	1+ or higher	4949	15.2%	3.62	1.97 (1.66 to 2.33)

ATRIA indicates Anticoagulation and Risk Factors in Atrial Fibrillation; CI, confidence interval; Ref, reference; M, male; F, female; Y, yes; N, no; ESRD, end‐stage renal disease.

Eight variables were chosen in >60% of the 1000 bootstrap sample models: age (chosen for all 1000 bootstrap samples), prior stroke (for all 1000 samples), female (for 999 samples), diabetes mellitus (for 849 samples), heart failure (for 704 samples), hypertension (for 703 samples), proteinuria (for 870 samples), and eGFR<45 mL/min per 1.73 m^2^/ESRD (for 624 samples) plus the age by prior stroke interaction term (for 856 samples). Of note, CAD and PAD did not meet the bootstrap sample selection threshold and were not significant predictors when added to the final model (Table 2). In the multivariable model, the hazard ratio for CAD was 1.10 (*P*=0.27) and for PAD was 0.92 (*P*=0.70).

**Table 2.** Regression Coefficients for the ATRIA Stroke Risk Model and Points Assigned for the ATRIA Stroke Risk Score

Clinical Characteristic	Coefficient Estimate	Hazard Ratio	Points Assigned
Age, y			
≥85, with prior stroke	2.48	11.92	9
75 to 84, with prior stroke	2.03	7.61	7
65 to 74, with prior stroke	2.07	7.89	7
<65, with prior stroke	2.20	8.99	8
≥85, without prior stroke	1.85	6.38	6
75 to 84, without prior stroke	1.33	3.79	5
65 to 74, without prior stroke	0.74	2.10	3
Female	0.42	1.52	1
Diabetes mellitus	0.34	1.40	1
Chronic heart failure	0.24	1.27	1
Hypertension	0.22	1.24	1
Proteinuria	0.34	1.40	1
eGFR<45 or ESRD	0.28	1.33	1

ATRIA indicates Anticoagulation and Risk Factors in Atrial Fibrillation; eGFR, estimated glomerular filtration rate; ESRD, end‐stage renal disease.

The c‐index for the final model was 0.74 (95% CI, 0.72 to 0.76) for the derivation two thirds of the cohort, which decreased to 0.72 (95% CI, 0.68 to 0.75) when the model was tested in the one‐third validation cohort. The goodness‐of‐fit statistic did not show evidence of poor calibration in either cohort (*P*=0.14 and *P*=0.78 for the derivation and the validation sets, respectively). To construct a stroke risk score, we assigned points to predictor variables proportional to the size of their regression coefficients in the model (Table 2). Age and prior stroke were the dominant risk predictors. Possible scores ranged from 7 to 15 for patients with a prior stroke and from 0 to 12 for those with no history of stroke (Table 3).

**Table 3.** ATRIA Stroke Risk Model Point Scoring System

Risk Factor	Points Without Prior Stroke	Points With Prior Stroke
Age, y		
≥85	6	9
75 to 84	5	7
65 to 74	3	7
<65	0	8
Female	1	1
Diabetes	1	1
CHF	1	1
Hypertension	1	1
Proteinuria	1	1
eGFR<45 or ESRD	1	1

Possible point scores range from 0 to 12 for those without a prior stroke and from 7 to 15 for those with a prior stroke. ATRIA indicates Anticoagulation and Risk Factors in Atrial Fibrillation; CHF, congestive heart failure; eGFR, estimated glomerular filtration rate; ESRD, end‐stage renal disease.

### Comparison of the ATRIA, CHADS_2_ and CHA_2_DS_2_‐VASc Risk Scores

Thromboembolic event rates by the 3 scoring systems for the entire ATRIA cohort are shown in Table 4. For the full range of point scores, the c‐index was 0.73 (95% CI, 0.71 to 0.75) for the ATRIA score, 0.69 (95% CI, 0.67 to 0.71) for the CHADS_2_ score, and 0.70 (95% CI, 0.68 to 0.72) for the CHA_2_DS_2_‐VASc score (Table 5, column B).

**Table 4.** Thromboembolic Event Rates by Point Score for Anticoagulation and Risk Factors in Atrial Fibrillation (ATRIA), CHADS_2_, and CHA_2_DS_2_‐VASc Risk Scores*

Points	ATRIA			CHADS_2_*			CHA_2_DS_2_‐VASc*		
	Events	Person‐Years	Rate per 100 Person‐Years	Events	Person‐Years	Rate per 100 Person‐Years	Events	Person‐Years	Rate per 100 Person‐Years
0	2	2652	0.08	22	6126	0.36	1	2493	0.04
1	12	2819	0.43	121	10 084	1.20	21	3806	0.55
2	14	1419	0.99	253	9757	2.59	46	5560	0.83
3	13	1780	0.73	178	4782	3.72	121	7305	1.66
4	19	2960	0.64	81	1309	6.19	193	6898	2.80
5	36	3614	0.99	19	450	4.23	175	4057	4.31
6	83	4346	1.91	11	101	10.84	85	1783	4.77
7	119	4768	2.50	—	—	—	24	498	4.82
8	151	3913	3.86	—	—	—	14	179	7.82
9	104	2400	4.33	—	—	—	5	30	16.62
10	75	1181	6.35	—	—	—	—	—	—
11	31	501	6.18	—	—	—	—	—	—
12	20	183	10.95	—	—	—	—	—	—
13	4	53	7.52	—	—	—	—	—	—
14	2	12	16.36	—	—	—	—	—	—
15	0	7	0	—	—	—	—	—	—
All	685	32 609	2.10	—	—	—	—	—	—

* Heavy black lines identify thresholds for low‐, moderate‐, and high‐risk categories for the 3 stroke risk point scores using published cut points.^8–9^

* The CHADS_2_ score assigns points as follows: 1 point each for the presence of congestive heart failure, hypertension, age ≥75 years, and diabetes mellitus and 2 points for history of stroke/transient ischemic attack.

* The CHA_2_DS_2_‐VASc score assigns points as follows: 1 point each for congestive heart failure/left ventricular dysfunction, hypertension, diabetes mellitus, vascular disease, age 65 to 74 years, and female sex and 2 points each for age ≥75 years and stroke/transient ischemic attack/thromboembolism.

**Table 5.** C‐Index Values for the ATRIA, CHADS_2_, and CHA_2_DS_2_‐VASc Stroke Risk Scores

A	B	C	D	E	F	G	H	I
Score	ATRIA: full cohort, all TE events	ATRIA: full cohort, severe TE events	ATRIA: primary prevention cohort	ATRIA‐CVRN: full cohort, all TE events	ATRIA‐CVRN: full cohort, severe TE events	ATRIA‐CVRN: primary prevention cohort	ATRIA: primary prevention cohort, severe events	ATRIA‐CVRN: primary prevention cohort, severe events
ATRIA, full score	0.73 (0.71 to 0.75)	0.76 (0.74 to 0.79)	0.71 (0.69 to 0.74)	0.70 (0.67 to 0.72)	0.75 (0.72 to 0.78)	0.69 (0.67 to 0.72)	0.75 (0.73 to 0.78)	0.74 (0.71 to 0.77)
CHADS_2_, full score	0.69 (0.67 to 0.71)	0.72 (0.70 to 0.75)	0.66 (0.64 to 0.69)	0.66 (0.64 to 0.69)	0.69 (0.66 to 0.72)	0.65 (0.63 to 0.68)	0.71 (0.68 to 0.73)	0.68 (0.65 to 0.71)
CHA_2_DS_2_‐VASc, full score	0.70 (0.68 to 0.72)	0.73 (0.71 to 0.75)	0.69 (0.67 to 0.71)	0.68 (0.66 to 0.70)	0.71 (0.68 to 0.74)	0.67 (0.65 to 0.69)	0.72 (0.69 to 0.74)	0.70 (0.68 to 0.73)
ATRIA, 3 categories	0.69 (0.67 to 0.71)	0.72 (0.70 to 0.74)		0.66 (0.64 to 0.68)				
CHADS_2_, 3 categories, published thresholds	0.66 (0.64 to 0.68)	0.70 (0.68 to 0.72)		0.65 (0.63 to 0.67)				
CHA_2_DS_2_‐VASc, 3‐categories published thresholds	0.58 (0.57 to 0.59)	0.59 (0.58 to 0.60)		0.58 (0.58 to 0.59)				

ATRIA indicates Anticoagulation and Risk Factors in Atrial Fibrillation; TE, thromboembolic.

**Figure 2.**
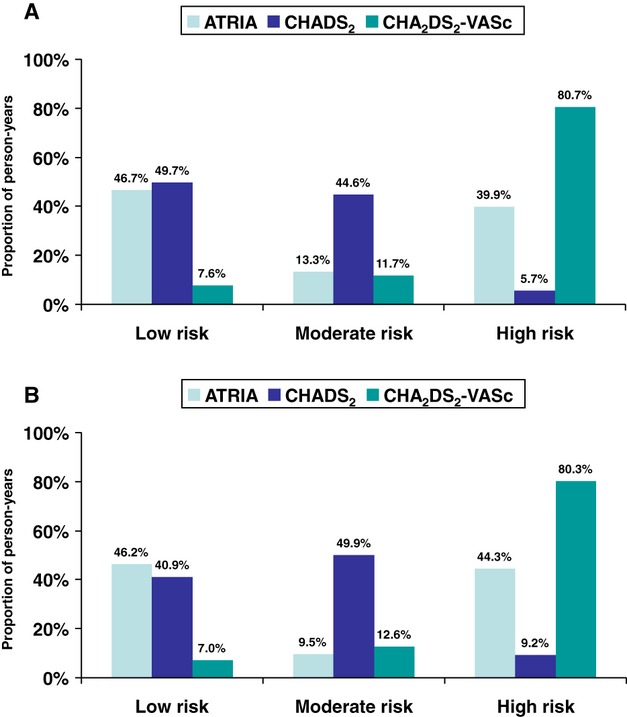
A, Distribution of person‐years by risk category applying the Anticoagulation and Risk Factors in Atrial Fibrillation (ATRIA), CHADS_2_, and CHA_2_DS_2_‐VASc stroke risk scores to the full ATRIA off‐warfarin cohort. Annual stroke rates for low‐, moderate‐, and high‐risk groups were 0.63%, 1.91%, and 3.89%, respectively, using the ATRIA score, 0.88%, 2.96%, and 5.97% with CHADS_2_, and 0.04%, 0.55%, and 2.52% with CHA_2_DS_2_‐VASc. B, Distribution of person‐years by risk category applying the ATRIA, CHADS_2_, and CHA_2_DS_2_‐VASc stroke risk scores to the ATRIA‐CVRN off‐warfarin cohort. Annual stroke rates for low‐, moderate‐, and high‐risk groups were 0.68%, 1.53%, and 3.22%, respectively, using the ATRIA score, 0.68%, 2.47%, and 4.10% with CHADS_2_, and 0.16%, 0.27%, and 2.29% with CHA_2_DS_2_‐VASc.

The ATRIA score was collapsed into low (0 to 5 points), moderate (6 points), and high (7 to 15 points) risk categories to fit annualized event rates of <1%, 1% to <2%, and ≥2% per year, respectively. The resulting c‐index was 0.69 (95% CI, 0.67, 0.71). By contrast, using the published low/moderate/high risk categories for the CHADS_2_ (0 to 1, 2 to 3, and 4 to 6 points, respectively) and the CHA_2_DS_2_‐VASc scores (0, 1, and ≥2 points, respectively), the resulting c‐indexes were 0.66 (95% CI, 0.64 to 0.68) and 0.58 (95% CI, 0.57 to 0.59), respectively (Table 5, column B). The low c‐index for the CHA_2_DS_2_‐VASc score reflected its very low threshold for its high‐risk category (Figure 2A).

The ATRIA score accurately categorized large fractions of person‐years as low (47%) or high (40%) risk (Figure 2A, Table 4). By contrast, using published thresholds, the CHADS_2_ score placed most person‐years in low‐risk (50%) and moderate‐risk (45%) categories and the CHA_2_DS_2_‐VASc score placed most person‐years (81%) in the high‐risk category. The net reclassification improvement (NRI) from CHADS_2_ categories to the ATRIA categories was 26% (95% CI, 20% to 32%), composed predominantly of correct up‐classification from moderate risk (Table 6). The NRI from CHA_2_DS_2_‐VASc categories to the ATRIA categories was 27% (95% CI, 23% to 31%), resulting exclusively from down‐classification of CHA_2_DS_2_‐VASc high‐ and moderate‐risk categories.

**Table 6.** NRI Values Comparing ATRIA 3‐Category Stroke Risk Score (Low/Moderate/High) With 3‐Category Stroke Risk Scores for CHADS_2_, and CHA_2_DS_2_‐VASc Using Published Thresholds

	NRI Compared With CHADS_2_ (95% CI)	NRI Compared With CHA_2_DS_2_‐VASc (95% CI)
ATRIA, full cohort		
All TE events	26% (20% to 32%)	27% (23% to 31%)
Severe TE events	26% (18% to 34%)	32% (27% to 36%)
ATRIA‐CVRN cohort		
All TE events	24% (17% to 31%)	25% (21% to 30%)
Severe TE events	33% (23% to 42%)	33% (29% to 38%)

A positive NRI value indicates superior performance by the ATRIA score as measured by the percentage of patients correctly reclassified. NRI indicates net reclassification improvement; ATRIA‐CVRN, Anticoagulation and Risk Factors in Atrial Fibrillation–Cardiovascular Research Network; CI, confidence interval; TE, thromboembolic.

### External Validation in the ATRIA‐CVRN AF Cohort

In the ATRIA‐CVRN cohort, 496 stroke or other thromboembolic events occurred (93.4% ischemic strokes) during the 26 263 person‐years of follow‐up off warfarin contributed by 25 306 patients, for a TE rate of 1.9% per year. The same pattern of age by prior stroke interaction was seen as in the ATRIA cohort (Figure 1B), although the TE rates in patients with a prior stroke and younger than 85 years were lower in the ATRIA‐CVRN cohort. All ATRIA score risk factors had strong significant univariate effects in ATRIA‐CVRN, although diabetes and eGFR were not significant in the multivariable model (data not shown). In ATRIA‐CVRN the c‐index was 0.70 (95% CI, 0.67 to 0.72) for the full‐range ATRIA score versus 0.66 (95% CI, 0.64 to 0.69) for CHADS_2_ and 0.68 (95% CI, 0.66 to 0.70) for CHA_2_DS_2_‐VASc (Table 5, column E). The distribution of low‐, moderate‐, and high‐risk categories were remarkably similar to that seen in the ATRIA cohort (Figure 2B). The c‐index deteriorated when the point scores were reduced to 3 categories, but the general pattern of performance seen in the ATRIA cohort persisted (Table 5, column E). The c‐index for the 3‐category CHA_2_DS_2_‐VASc score was again quite low, at 0.58 (95% CI, 0.58 to 0.59). The pattern of NRI values favoring the ATRIA score that was seen in the original cohort was reproduced in the ATRIA‐CVRN cohort (Table 6).

### Risk Score Performance Predicting Severe Outcome Events

Of the 685 TE events in the ATRIA data set, 399 were severe (Rankin score ≥3 at discharge or the patient died within 30 days after the event). When the full point scores were applied to this subset of severe events, the c‐index increased to 0.76 (95% CI, 0.74 to 0.79) for the ATRIA score, 0.72 (95% CI, 0.70 to 0.75) for CHADS_2_, and 0.73 (95% CI, 0.71 to 0.75) for CHA_2_DS_2_‐VASc (Table 5, column C). The NRI values for the 3‐category scores with outcomes restricted to severe events were similar to those for all outcome events (Table 6).

We tested the 3 full‐range scores on the subset of 282 severe events in the ATRIA‐CVRN cohort. The c‐index for the ATRIA score was 0.75 (95% CI, 0.72 to 0.78), in excellent agreement with the derivation cohort result. By contrast, in the ATRIA‐CVRN cohort the c‐index deteriorated more for the CHADS_2_, 0.69 (95% CI, 0.66 to 0.72), and CHA_2_DS_2_‐VASc, 0.71 (95% CI, 0.68 to 0.74) scores (Table 5, column F). NRIs comparing the 3‐category ATRIA score with those of CHADS_2_ and CHA_2_DS_2_‐VASc were very similar to the values seen with the original ATRIA cohort (Table 6).

### Prediction in the Primary Prevention Subset of Patients

Focusing only on cohort members without a history of prior stroke (primary prevention), the c‐index for the full‐range ATRIA score was 0.71 (95% CI, 0.69, 0.74) versus 0.66 (95% CI, 0.64, 0.69) for CHADS_2_ and 0.69 (95% CI, 0.67, 0.71) for the CHA_2_DS_2_‐VASc score (Table 5, column D). The same order was preserved in the ATRIA‐CVRN cohort with modest reductions in the c‐index (Table 5, column G). When the primary prevention analysis was restricted to severe outcomes, the c‐index improved (Table 5, column H and I). Agreement across the 2 cohorts was particularly good for the ATRIA score.

### Risk Score Comparison Using Risk Category Thresholds Optimized for the ATRIA Cohort Data Set

We also tested point score cutoffs for the CHADS_2_ (0, 1, 2 to 6 points) and CHA_2_DS_2_‐VASc (0 to 2, 3, 4 to 9 points) scores that were optimized to fit the 1% and 2% per year thresholds for rate of thromboembolic (TE) events that we used in categorizing the ATRIA score (Table 4). Using these optimized cut points, the c‐index for the 3‐category CHA_2_DS_2_‐VASc score improved markedly to 0.68 (95% CI, 0.66 to 0.69) but was still lower than the c‐index for the ATRIA score. The c‐index for the CHADS_2_ score was largely unchanged, at 0.65 (95% CI, 0.64 to 0.67).

Using these optimized cut points, the net reclassification improvement (NRI) for the ATRIA 3‐category score was 20% (95% CI, 17 to 24) versus the CHADS_2_ score and 8.6% (95% CI, 5.3 to 12) versus the CHA_2_DS_2_‐VASc score. These lower NRI results indicate less benefit from using the ATRIA score than had been observed with the published cut points. However, the ATRIA score still resulted in net correct reclassification compared with both the CHADS_2_ and the CHA_2_DS_2_‐VASc scores.

## Discussion

Anticoagulants are highly effective in preventing ischemic stroke in persons with AF, but they can also cause major hemorrhage. Although prediction of risk of both ischemic stroke and major bleeding is relevant to the anticoagulation decision, formal decision analyses indicate that for most patients with AF, risk of ischemic stroke is the more important one.^25–26^ Improved prediction of stroke risk in patients with AF would allow better targeted anticoagulant therapy. We used the large observational follow‐up of ATRIA AF cohort members not taking warfarin to optimize use of common clinical features to predict stroke risk. We validated the core risk factors used in the CHADS_2_ score but added features that we have previously reported to predict stroke (ie, female sex, renal dysfunction, and proteinuria).^14,16^ Most importantly, we used a broader range of age categories, a decision consistent with multiple prior reports.^3,27^ We found strong amplification of stroke risk across the entire age range, with individuals aged ≥85 years at nearly double the risk of those aged 75 to 84 years. However, individuals who had had a stroke were at elevated risk regardless of age. Age, prior stroke, and their interaction were the dominant risk factors in our model. As with multiple prior studies, we did not observe significant incremental risk prediction from coronary disease, the major component of “VASc” in the CHA_2_DS_2_‐VASc score.^9–10,28^ Our resulting ATRIA risk scheme performed better among ATRIA cohort members than did the CHADS_2_ and CHA_2_DS_2_‐VASc schemes, which are recommended in leading clinical guidelines.^4–7^ The c‐index was greater, and there was positive net reclassification improvement. From a practical point of view, more patients were accurately classified as low or high risk. Indeed, 46% of patients in both the derivation and validation ATRIA cohorts were categorized by the ATRIA score as having <1% per year risk. Such low risk indicates a small net clinical benefit from anticoagulation.^23,27^ Importantly, all aspects of improved performance with the ATRIA score were reproduced in the separate, larger, and contemporary ATRIA‐CVRN cohort of incident AF patients.

When we restricted outcome to severe events, model discrimination improved, resulting in a c‐index of 0.76 for the ATRIA cohort. Clinically, prevention of severe events is more important than prevention of minor events and accurate prediction of severe events more relevant to the anticoagulation decision. Our findings should prompt other investigators developing stroke risk models for AF to also focus on severe outcomes.

All AF patients who have had an ischemic stroke are at high risk of future stroke, and anticoagulation is strongly indicated. Risk prediction in primary prevention is more uncertain and where most of the challenge of anticoagulation decision‐making lies. Our ATRIA model provides a separate scoring scheme for primary prevention, and this scheme also performed better than the comparator schemes, a finding that was replicated in the separate, external ATRIA‐CVRN cohort. Performance of the ATRIA score was particularly good in predicting severe events in the primary prevention cohort.

Recent reports highlight the promising performance of biomarkers in predicting stroke in patients with AF.^29^ Our ATRIA score appears to provide an improved clinical risk factor model on which to add such biomarkers with the goal of optimal risk prediction.

Our model development had notable strengths. We based our findings on a generalizable community‐based cohort with a wide range in age and comorbidities representative of patients treated in usual care settings. The study sample was large, with hundreds of physician‐validated outcome events, allowing powerful assessment of the limited number of candidate predictor variables we tested. Case record review allowed us to assign Rankin severity ratings. We employed a split‐sample derivation/validation approach, and we used multiple bootstrap sample model‐building to reduce overconfidence in the selection of predictors. Most importantly, we validated our model and its relative performance versus the 2 leading alternative risk scores in an independent, contemporary, “external” validation cohort—the ATRIA‐CVRN inception AF cohort. Testing in additional data sets will assess the broader generalizability of our findings. Although the ATRIA score is more complicated than commonly used schemes, particularly CHADS_2_, the increasing availability of automated real‐time decision support tools based on data routinely available in electronic medical records will likely reduce the need for a simple scoring system.

## Conclusion

The ATRIA stroke risk score for AF patients was rigorously developed in a community‐based cohort and externally validated in a separate recently assembled community‐based AF cohort. Our results highlight the importance of including multiple categories of age, prior stroke, and their interaction in determining stroke risk in AF patients. The ATRIA score's performance was superior to the widely used CHADS_2_ and CHA_2_DS_2_‐VASc risk schemes in terms of c‐index and NRI. It identified a substantially larger fraction of patients at low stroke risk. Its performance was particularly good in predicting severe strokes, the category of stroke we are most interested in avoiding. Finally, the ATRIA score lends itself to calculating risk in primary prevention patients, the large group whose stroke risk is most uncertain and for whom personalizing the anticoagulation decision is most pressing. The ATRIA score provides an improved clinically based model on which to add informative biomarkers.
